# Implementation of free maternal and child healthcare policies: assessment of influence of context and institutional capacity of health facilities in South-east Nigeria

**DOI:** 10.1080/16549716.2018.1535031

**Published:** 2018-10-24

**Authors:** Daniel C. Ogbuabor, Obinna E. Onwujekwe

**Affiliations:** a Department of Health Administration and Management, University of Nigeria Enugu Campus, Enugu, Nigeria; b Department of Health Systems and Policy, Sustainable Impact Resource Agency, Enugu, Nigeria; c Health Policy Research Group, Department of Pharmacology and Therapeutics, College of Medicine, University of Nigeria Enugu Campus, Enugu, Nigeria

**Keywords:** Nigeria, capacity of health facilities, management of health facilities, free healthcare, policy implementation

## Abstract

**Background**: Studies examining how the capacity of health facilities affect implementation of free healthcare policies in low and middle-income countries are limited.

**Objective**: This study describes how the context and institutional capacity of health facilities influenced implementation of the free maternal and child health programme (FMCHP) in Enugu state, South-east Nigeria.

**Methods**: We conducted a qualitative case study at the state level and in two health districts (Isi-Uzo and Enugu Metropolis) in Enugu State. Data were collected through document review and semi-structured, in-depth interviews with policymakers (n = 16), healthcare providers (n = 16) and health facility committee leaders (n = 12) guided by an existing capacity framework and analysed using a thematic framework approach.

**Results**: The findings reveal that active health facility committees, changes in provider payment process, supportive supervision, drug revolving fund, availability of medical equipment, electronic data transmission and staff sanction system enhanced the capacity of health facilities to offer free healthcare. However, ineffective decentralisation, irregular supervision and weak citizen participation limited this capacity. Uncertain provider payment, evidence of tax payment policy and a co-existing fee-exempt scheme constrained health facilities in following the FMCHP guidelines. Poor recording and reporting skills and lack of support from district officials constrained providers’ adherence to claims’ submission timeline. Poor funding, weak drug supply system, inadequate infrastructure and lack of participatory decision-making constrained delivery of free healthcare. Insufficient trained workforce, mission-inconsistent postings and transfers, and weak staff disciplinary system limited the human resource capacity.

**Conclusions**: Effectiveness of FMCHP at the health facility level depends on the extent of decentralisation, citizen participation, concurrent and conflictive policies, timely payment of providers, organisation of service delivery and human resources practices. Attention to these contextual and institutional factors will enhance responsiveness of health facilities, sustainability of free healthcare policies and progress towards universal health coverage.

## Background

Many low and middle-income countries (LMICs) have adopted free healthcare policies to mitigate the negative impact of user fees on the poor, improve access to health services and accelerate progress towards universal health coverage and the sustainable development goals (SDGs) [1–3]. That notwithstanding, poor use of public health facilities and poor health indicators in low resource settings persist [3]. Only 9.5% of the countries with maternal mortality ratios above 100 deaths per 100,000 live births in 1990 achieved the millennium development goal (MDG) target of three-quarter reduction in maternal mortality [4]. Of the 104 LMICs, only about 23% attained the MDG4 target to reduce under-five mortality by two-thirds relative to 1990 levels [4]. In Nigeria, despite adoption of free maternal and child healthcare policies in 2006 [5], reduction in maternal and childhood mortality rates have been lower than expected [6]. Although capacity of health facilities, referred to as ability to perform appropriate tasks effectively, efficiently and sustainably [7,8], affect the effectiveness of free healthcare policies, theory-driven studies examining the effect of capacity of health facilities on implementation of user fee removal policies are limited.

Growing evidence suggests that factors affecting the capacity of health facilities to implement free healthcare policies are context-sensitive. Use of service charters to curb informal payments to service providers [9], active health facility committees (HFCs) including financial management [10–12], timely payment of providers [13], regular and uninterrupted drug supply [13], availability of clear guidelines on use of user fee replacement grants [14], an effective staff sanction framework, which reduces health worker absenteeism [15], have enabled health facilities to effectively implement free healthcare policies. In contrast, barriers to health facilities implementing free healthcare policies include weak decentralization [10,16–18]; poor supervision and monitoring [19,20]; weak referral systems [17]; absence of written implementation guidelines [14,21]; shortage of staff and inconsistent recording and reporting [10,20]; low social accountability and community involvement [9,22–24]; delayed payment of providers [12,21,25]; lack of adequate drug stock [17,18,24,26–31]; mistrust between providers and patients [19,32–34]; absenteeism [10,15,20]; poorly motivated health workers [18,24]; lack of financial incentives [35]; preference for urban postings [19,36]; funding inadequacy in health facilities [16]; inadequate physical infrastructure [18,24,36]; unavailability of guidelines for use of user fee replacement fund [37] and unclear procedures for targeting beneficiaries [3].

Enugu State adopted the free maternal and child healthcare programme (FMCHP) in 2007, which removed user fees at the point of service delivery for pregnant women and under-five children based on a minimum services package [38]. The FMCHP, which is tax-funded through State and Local Government contributions, is delivered mainly through public primary and secondary health facilities. Basic child healthcare services including health education, growth monitoring, immunization, nutritional supplementation, de-worming and integrated management of childhood illnesses; basic ante-natal care, safe deliveries, post-natal care, family planning services, routine laboratory investigations and basic emergency obstetric care are provided across the two levels of care. Additionally, the cottage and district hospitals provide comprehensive maternity services and treat difficult childhood illnesses. The state teaching hospital provides only referral care.

**Figure 1.**
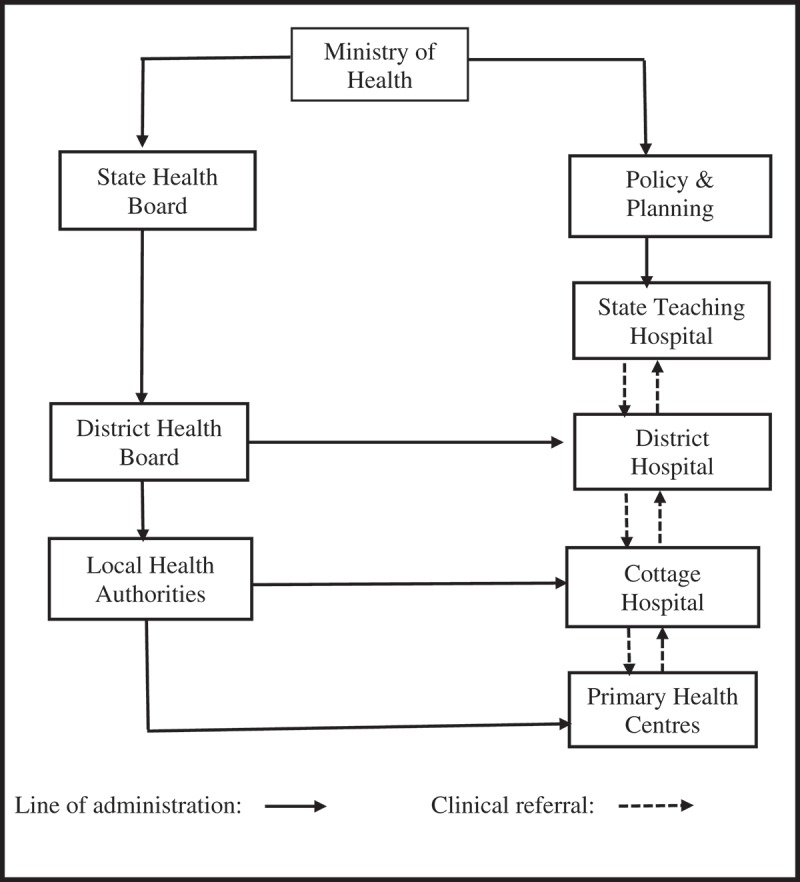
Enugu State decentralized health system.

The FMCHP is administered through a decentralized health system in which the State Ministry of Health (MOH) is organized into a Policy Development and Planning Directorate (PDPD) and State Health Board (SHB); and the state delineated into seven District Health Boards (DHBs) and 68 Local Health Authorities (LHAs) (Figure 1). The PDPD, through a Steering Committee, governs the FMCHP and manages FMCHP funds. The SHB, through an Implementation Committee, oversees the DHBs and supervises district-level health workers, vets free care claims and pays health facilities for patients who receive free services. The DHBs monitor implementation of FMCHP at the LHAs and district hospitals; ensure regular and uninterrupted supply of FMCHP forms to health facilities; and collate facility claims from LHAs and submit them to the SHB. The LHAs monitor implementation of the FMCHP in all primary health facilities and cottage hospitals. LHAs support health facilities to prepare claims and submit completed claims to the DHBs. Health facilities are expected to provide responsive services, document all FMCHP expenditures, completely fill and promptly submit claim forms, track submitted claims, report major service delivery gaps to government and handle minor infrastructural gaps.

Evidence indicates that the initial improvement on service utilization has not been sustainable, out-of-pocket payments are common, and the FMCHP does not seem pro-poor nor does it improve use of the public health system [39–42]. The maternal mortality rate in Enugu is 645/100,000 live births [43], higher than the national rate of 576/100,000 live births [44]. Under-five mortality rate in South-east Nigeria, including Enugu State, is 131/1000 live births; slightly higher than national average of 128/100,000 live births [44]. Only 45% of under-five children are fully vaccinated while 8.6% of the children have no vaccination at all in Enugu State [44]. Delivery at public health facilities in Enugu State, though improved by 15.1% from 2008 to 2013, is still low at about 36.5% [44]. Yet, little is known about facility-level factors affecting implementation of the FMCHP.

The purpose of this study was to examine how context and institutional capacity of health facilities affect implementation of FMCHP in Enugu, South-east Nigeria. Such evidence will help decision makers in Nigeria and similar settings to develop a governance model for strengthening the capacity of health facilities to offer free healthcare that contributes towards universal health coverage and the SDGs.

## Methods

### Conceptual framework

**Figure 2.**
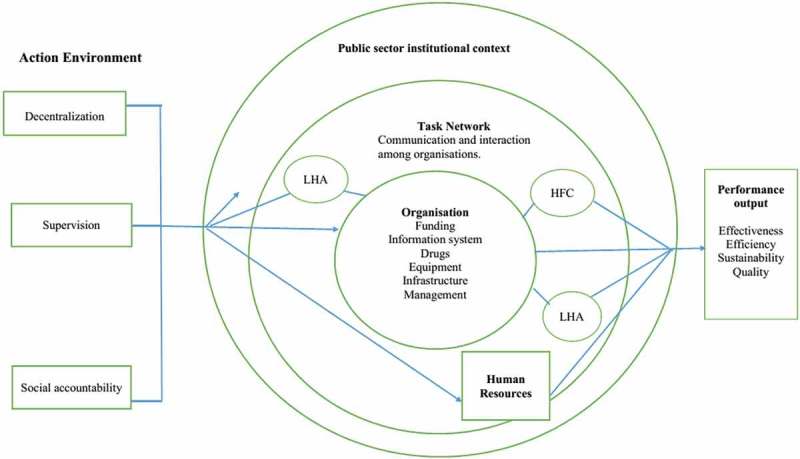
Conceptual Framework of the study. Source: Adapted from Hilderbrand and Grindle institutional capacity framework (1997).

The study was guided by the Hilderbrand and Grindle (1997) Capacity Framework [45], comprising five dimensions – action environment, institutional context of public sector, task network, organisation and human resources – that impact on public service performance as shown in Figure 2. Action environment focuses on the external context of health facilities such as decentralization, supervision and citizen participation. Institutional context of public providers deals with general rules, procedures and concurrent policies that shape performance of health facilities. Task network entails relations among actors involved in implementing FMCHP. Organisation encompasses structures and processes in health facilities that enhance service delivery including physical resources, information systems, drug delivery systems, and finances. Human resources comprise attraction and retention of individuals who work in public health facilities. Optimal capacity of health facilities would result in responsiveness of service delivery.

### Study setting

This study, which is part of a doctoral thesis that assessed the governance of FMCHP in Enugu State, Nigeria [46], took place at the MOH and in two districts (Isi-Uzo and Enugu Metropolis) of Enugu State, South-east Nigeria. In 2017, Isi-Uzo and Enugu Metropolis had 209,872 and 1,021,912 populations, respectively, out of which women of childbearing age constitute 42.8% and 46.9%, respectively [44]. Children under five years represent 18.1% and 15.5%, respectively, of the population of Isi-Uzo and Enugu Metropolis [44]. The two districts have, each, a DHB, four LHAs, a general hospital and a network of cottage hospitals and primary health facilities. Many health facilities have health facility committees (HFCs) allowing for community involvement in health facility management.

### Research design

This study adopted a qualitative, case study design because experiences with implementation of the FMCHP are embedded in the context that formed the focus of inquiry [47,48]. The case is the FMCHP of Enugu State in South-east Nigeria. Two districts were selected as embedded case study sites. The phenomenon of inquiry is the implementation of the programme.

### Study population and sampling strategy

We categorised the seven health districts into two groups of three well-performing and four less-well-performing districts using provider payment data over five years (2009–2014), which ranged from 2% to 26% with a median of 14% serving as the cut-off point [46,49]. We used provider payment data for the categorisation, because the health information management system does not disaggregate maternal and child health utilisation by user fee and fee-exempt. Enugu Metropolis and Isi-Uzo districts were each randomly selected from the well-performing and less-well-performing districts. The districts were selected to maximise the diversity of implementation experiences and understand how contextual and institutional factors influenced the capacity of health facilities to offer free MCH services in two contrasting settings. We purposively selected policymakers (n = 16), health care providers (n = 16) and health facility committees (HFC) leaders (n = 12) because of their positions, involvement in FMCHP implementation and willingness to participate in the study. In each district, healthcare providers were selected from the district hospital, the busiest cottage hospital and six primary health centres that have active HFCs. Key public officials at state and district levels were used as gatekeepers to identify participants.

### Data collection methods

A semi-structured, in-depth interview guide, developed based on conceptual framework of this study, validated by two experts and adapted following analysis of pilot interviews, was used to conduct interviews. In total, 44 people were interviewed between February and September 2015 (Table 1) using the same interview guide. The guide explored how decentralisation, monitoring and supervision, citizen participation, concurrent policies, provider payment procedures, and resource availability in health facilities affected implementation of FMCHP (Appendix 1). Appointments were sought by cell phone or personal visits. Participants were interviewed in their offices or health facilities. Interviews, which lasted about 60–90 min, were conducted in English, audiotaped, transcribed verbatim and transcripts sent back to participants to confirm the credibility of the transcripts [50].

**Table 1.** Socio-demographic profile of the participants.

Participants	Location	Post	Total number	Male	Female
Policymakers	State Ministry of Health (Policy Development and Planning Directorate)	Steering Committee members	5	3	2
	State Health Board	State Implementation Committee members	5	5	
	District Health Boards	District Chief Executive Officers	2	2	
	Local Health Authorities (LHA)	LHA Secretaries	4	4	
Providers	District A	Head of facilities	8	2	6
	District B	Head of facilities	8	1	7
Citizens	District A	Health Facility Committee Leader	6	5	1
	District B	Health Facility Committee Leader	6	6	

Additional data were collected through document review. Thirteen documents, relevant to the research questions, were identified in consultation with key government officials (Appendix 2). The documents reviewed included policy documents, programme reports and memoranda on FMCHP.

### Data analysis

Data were analysed using a framework approach which involved coding the data, mapping and organising data under common themes, and interpretation [51]. NVivo 11 software was used for coding and categorising the data. Codes were developed from the conceptual framework and reading the data. Coding was done by two persons guided by a coding framework, which enhanced validity and reliability of this study [52]. Data analysis was piloted with six transcripts and all differences in coding were reconciled before full data analysis. The findings from different sources were compared for patterns of convergence and divergence. Furthermore, the findings of this study were discussed with study participants and key health systems stakeholders in a validation meeting.

### Ethical considerations

The Health Research Ethics Committee of the University of Nigeria Teaching Hospital Enugu, Nigeria, approved this study. All participants provided written informed consent.

## Results

Five themes emerged from the study, namely action environment (three sub-themes), institutional context of public providers (three sub-themes), task network (two sub-themes), organisation (four sub-themes) and human resources (three sub-themes). Each sub-theme is further divided into two categories (enabling factor and constraints) as shown in Table 2.

**Table 2.** Factors influencing capacity of health facilities to provide free maternal and child health services in Enugu State, Nigeria.

Theme	Sub-themes	Enabling factors	Constraints
Action environment	Decentralization	District Health System policy	Dysfunctional District Health Boards and Local Health Authorities
			Non-existent District FMCHP Committees
	Supervision	Integrated supportive supervision. Availability of vehicles	Weak supervision and monitoring of provider performance by district officials due to irregular overheads.
	Accountability	Active HFCs that monitor drug availability and staff attendance	Health facilities lack complaint box and service charters
		HFCs also resolve users’ complaints	
Institutional context of the public sector	Provider payment		Uncertain reimbursement procedure
	Concurrent policies		Concurrent evidence of tax payment policy
		SURE-P fee-exempt MCH services provided alternative to FMCHP in district A.	Non-harmonisation with federal-led SURE-P FMCHP
	Remuneration of health workers		Inadequate compensation of health workers
Task network	Awareness of benefits		Poor communication of entitlements and obligations to users
	Preparation and submission of claims		Lack of recording and reporting skills
			Non-involvement of health facility committees in claims reporting
			Lack of support from district and LHA officials in claims reporting
Organisation	Financial management	Policy of remitting 70% of approved service claims directly to health facilities.	Unpredictable remittance of 70% of FMCHP service expenditure to providers
			Insufficient overhead to maintain health facilities
			Unwillingness of providers to disclose financial information to HFC members
	HMIS	Availability of data collection tools Use of mobile phones for data transmission.	Poor funding of monitoring and evaluation activities Use of mobile phones for data transmission by-passes District Information Team
			Absence of data review meetings
	Drugs	Approved drug claims remitted to health facilities through central medical stores	Collapse of drug revolving fund due delayed/non-payment of providers
			Health workers dispense private drugs
			Supply of drugs nearing expiry or beyond scope of services
	Infrastructure		Poor physical infrastructure to meet service entitlements
		Equipment donated by development partners	Equipment is packed in stores
			Lack of ambulance to support referrals Insecure working environment
Human resources	Availability	Presence of SURE-P staff	Shortage of health workers, worse in rural health facilities
	Posting & transfer		Postings and transfers to less busy or urban facilities
	Disciplinary procedure	Existence of sanction procedure. HFCs monitor staff attendance	Weak enforcement of sanction High rate of absenteeism among health workers

### Action environment of providers

Decentralisation, supervision and accountability emerged as key themes in the action environment.

### Decentralisation

Despite the existence of district health system (DHS) policy, most policymakers and providers identified dysfunctional District Health Boards (DHBs) and District FMCHP Committees as constraints to providers’ capacity to offer free health care. Meetings of DHBs were rarely held. Quarterly review meetings that provided opportunities for review of FMCHP implementation were no longer held since 2012 due to irregular funding (DR7, DR10). A few policymakers and providers observed that Local Government monitoring and evaluation officers by-pass the district information office in data transmission from health facilities to the MOH. Most HFC leaders do not know about the DHBs’ activities, nor the existence of the District FMCHP Committee.

### Supervision

Most policymakers and providers mentioned that supervision is supportive and offers opportunity for on-the-job capacity building, but it is irregular. In Isi-Uzo, all HFC leaders revealed that supervision by government officials is poor. ‘One hardly sees supervisors from the local government. The occasional supervisors we see are those of SURE-P and PATHS2 (development partners)’ (HFC leader 4). Some HFC leaders in Enugu Metropolis mentioned that providers receive routine quarterly supervision. Conversely, some HFC leaders in Enugu Metropolis stated that ‘they (supervisors) do not even visit most of the health centres that are within accessible areas’ (HFC leader 11). Though vehicles were provided to districts to enhance supervision, the districts lack operating funds to conduct regular supervision (DR10).

### Accountability

All categories of participants identified HFCs as the only functioning social accountability initiative in health facilities. HFCs effectively oversee the Drug Revolving Fund (DRF) and staff attendance. ‘We have been monitoring health workers. Their salaries were not paid until we endorsed their time book’ (HFC leader 6). HFCs are not involved in identifying eligible users of free care and managing free care refunds (DR9). HFCs lack the legislative framework for effective and efficient discharge of their functions (DR2). Some policymakers and HFC leaders noted that HFC Alliances exist but are ineffective. Most policymakers, providers and HFC leaders corroborated the findings that health facilities lacked service charters and complaint boxes (DR4, DR5). A few policymakers and providers observed that complaint boxes and service charters may not be useful because ‘people know their right…they are ready to bare their mind any time anywhere, even if it hurts you’ (Provider 8).

### Institutional context of providers

Reimbursement standard, concurrent policies and poor remuneration emerged as key themes related to the institutional context of providers.

### Reimbursement standard

Reimbursement was often delayed, health facilities were sometimes paid fractions of their claims, and health workers did not seem to understand the reimbursement procedure (DR2, DR3). Most policymakers and providers indicated that reimbursement of health facilities was poorly enforced in both districts. Many health facilities were not paid for over a year resulting in stoppage of free maternal and child health services in Isi-Uzo. In Enugu Metropolis, delayed reimbursement was compounded by under-payment of providers which resulted in interrupted free maternal and child health services. Delayed provider payment resulted in stock-outs and resumption of user fees (DR2, DR5, DR7, DR10, DR11, DR12). In both districts, most HFC leaders were not aware of the reimbursement process but a few HFC leaders corroborated policymakers’ and providers’ views that many providers no longer submitted claims for reimbursement.

“Ooh! There was a time the fund ceased for almost a year or two years…. the officers-in-charge lost hope, almost all of them lost hope. I do not even think that most providers are submitting their invoices now from this district” (Policymaker 14).

### Concurrent policies

Evidence of tax payment was introduced as a proof of residence in the state but resulted in under-utilisation of free services (DR1, DR2). Most district-level policymakers and providers incorrectly interpreted evidence of tax payment as tax clearance or a salary slip of a ‘civil servant’ husband, payment of some money in lieu of tax clearance or Gen 35 (evidence of appointment) for civil servants. The evidence of tax payment policy was never implemented in Isi-Uzo but partially implemented in Enugu Metropolis. Most HFC leaders in both districts were not aware that evidence of tax payment was implemented in their facilities. Few HFC leaders observed that, other than civil servants, clients pay some money in lieu of tax in Enugu Metropolis.

Most providers and HFC leaders in Isi-Uzo stated that Subsidy Reinvestment Programme (SURE-P), a federal government scheme, provided an alternative to the failing state-owned FMCHP. ‘Many patients now prefer going to SURE-P centre for treatment and delivery, where they did not have to pay out-of-pocket. Since then we rarely had patients here’ (Provider 8). The SURE-P supported health facilities with free drugs, delivery kits, equipment, health workers and infrastructure. However, few HFC leaders noted that the health workers ‘came only when we had the SURE-P intervention’ (HFC leader 4).

### Poor remuneration in the public sector

Participants of all categories indicated that non-implementation of nationally approved health salary scales, discriminatory compensation for different cadres of health workers and unpaid salaries undermine staff motivation to implement FMCHP:

“Though doctors were paid with approved salary scale, other health workers were not. It is not possible that other health workers will wholeheartedly run the free maternal and child healthcare programme per guidelines” (Provider 11).

### Task network of providers

Submission of claims and communication of service entitlements and obligations emerged as two main tasks of providers (Table 2). Most policymakers and providers stated that providers stopped submitting claims for reimbursement in Isi-Uzo, whereas providers in Enugu Metropolis delayed in submitting providers’ claims. Providers are not able to appropriately fill the claim forms and this was corroborated by document review (DR2, DR12). Few policymakers and most providers indicated that providers lacked support from district-level officials in preparation and submission of provider claims. ‘Our Local Health Authority does not support us very well. One will suffer, suffer and suffer before they sign claim form for one’ (Provider 2). The policymakers and providers stated that district-level officials withdrew from monitoring health facilities when health facilities were paid directly instead of through the district structures

Most HFC leaders in both districts said that they were not involved in claims preparation and submission. Only one HFC leader in Enugu Metropolis was aware of the reimbursement procedure. The HFC leader noted that providers were unable to correctly fill claims forms, and sometimes inflated their claims. Moreover, most policymakers, providers and HFC leaders agreed that providers do not sufficiently communicate service entitlements and obligations to users, which resulted in low awareness of the requirement for evidence of tax payment among users.

### Organisational factors

Facility funding, health management information system, drugs, equipment, infrastructure and communication shaped provider capacity.

### Facility funding

The FMCHP policy stipulates that 70% of health facilities’ claim for free services should be paid to the health facilities as operating cost (DR1). However, health facilities had poor access to the funds paid through the LHAs prior to 2010 when payment policy changed to direct facility funding (DR4, DR7, DR8). Most policymakers stated that the operating costs are paid directly to health facilities due to leakages at the LHAs. In Isi-Uzo, most district policymakers and providers observed that ‘health facilities no longer receive overhead from FMCHP due to cessation of reimbursement’ (Policymaker 14) and providers relied on funds from non-free services retained. One provider observed that ‘there was a time, health facilities received overhead, which was not regular, though. Within the preceding two years, I don’t think that there is anything like overhead for us’ (Provider 3). In Enugu Metropolis, most policymakers and providers indicated health facilities received insufficient reimbursements. The majority of HFC leaders in both districts indicated that they were signatories to health facilities’ account, but providers were unwilling to involve them in financial management.

### Health management information system (HMIS)

The capacity of providers to record and report data is low (DR3). However, most policymakers and providers in both districts indicated that use of mobile phones facilitated data transmission to the State HMIS office. Few policymakers and providers in both districts observed that data collection and transmission are poorly funded. ‘It was agreed that M&E officer should be given N10,000 every month, but it has not been implemented’ (Policymaker 13). Most providers in both districts also stated that inadequate staffing and absence of data review meetings constrained providers’ capacity to report and use data for planning. Most HFC leaders stated that HFCs are not involved in data collection and reporting. Few HFC leaders noted that charts of maternal and child healthcare attendance are discussed in their HFC meetings.

### Drugs, equipment and infrastructure

Existence of the drug revolving fund (DRF) scheme and procurement of drugs from the central medical stores enabled health facilities to offer free MCH services (DR2). Nonetheless, delayed payment of health facilities resulted in stock-outs (DR2, DR5, DR7, DR10, DR12). Most policymakers, providers and HFC leaders observed that providers’ DRF stocks were depleted due to delayed – or non-reimbursement of – FMCHP drug expenditure. Most policymakers and providers noted that many providers were supplied drugs beyond their level of care and drugs close to expiry. In both districts, most policymakers and few HFC leaders revealed that health workers dispensed private drugs in public health institutions. ‘The woman (provider) has resorted to buying drugs with her own money and selling them to those who come to the facility’ (HFC leader 4).

Most policymakers, providers and HFC leaders in both districts also stated that providers have equipment but lacked buildings to house equipment and support service delivery. Equipment were not always tailored to the scope of health facilities and staff received no training on how to operate and maintain them (DR2, DR3). Furthermore, in Isi-Uzo, few providers and HFC leaders highlighted the absence of a functional ambulance to support referral services. Even though ambulances were allocated to the district hospitals or the apex hospital in each local government (DR1), ambulances are usually used for other purposes and out of reach of the health facilities (DR3, DR10). In Enugu Metropolis, providers indicated that an insecure working environment led to incidence of thefts and sexual harassment of health workers on night shift. Many health facilities have dilapidated buildings, no perimeter fencing, poor electricity supply and poor water supply (DR2, DR3, DR5).

### Providers-clients’ trust and communication

Despite policy stipulation that users be educated on service entitlements and obligations in FMCHP (DR1), users’ lack of confidence in public health facilities limit use of free services (DR13). Whereas some policymakers and providers claimed that providers ‘delivered services in a professional manner’ (Policymaker 15), other policymakers and providers said that users complain about poor attitudes of healthcare providers. ‘Last time, I met a woman who said that she would never go for free MCH services because if one went to the health centre, providers would be insulting one’ (Provider 10). Most HFC leaders in Isi-Uzo stated that providers were not courteous and responsive to clients’ expectations; and are not available for afternoon and night shifts. Conversely, most HFC leaders in Enugu Metropolis said that providers were approachable and treated clients with respect.

### Human resources

Availability, posting and transfer, and weak disciplinary mechanisms emerged as sub-themes in human resources.

### Availability of health workers

Health facilities lack the appropriate number and mix of human resources (DR2, DR3, DR6). Most policymakers and providers indicated that lack of qualified health workers hampered delivery of free maternal and healthcare services. ‘We cried out bitterly that trained health workers in the system were too small to carry out this laudable programme.’ (Policymaker 16). Some providers and few HFC leaders observed that SURE-P improved availability of staff in Isi-Uzo.

### Posting and transfer

Participants of all categories supported the findings from document review that distribution of health workers was skewed in favour of urban districts (DR2, DR3, DR6). A few providers in Isi-Uzo observed that health workers were posted to health posts leaving busier health centres. In Enugu Metropolis, few providers stated that ‘some doctors were posted to less busy hospitals avoiding busier hospitals’ (Provider 12).

### Weak disciplinary mechanisms

Most policymakers stated that district officials do not sanction primary health staff and heads of facilities are reluctant to implement staff disciplinary procedures because ‘higher authorities’ (Policymaker 11) do not act on their recommendations. Also, whereas the Local Government Service Commission (LGSC) is responsible for managing health workers, the district officials have only technical oversight of primary healthcare service delivery. In both districts, most providers and HFC leaders explained that heads of health facilities do not comply with staff disciplinary procedures because ‘heads of facilities do not want to be marked as the ones responsible for the ordeal of their co-workers’ (Provider 7) and due to political interference. ‘Most staff, who are truant, are people that you are unable to discipline. When you query them, a higher authority will query you’ (Provider 15). Most HFC leaders stated that ‘no head of facility has actually queried or reported staff for insubordination’ (HFC Leader, 4).

## Discussion

This study has explored how the context and institutional capacity of health facilities affect implementation of the FMCHP. Evidence from this in-depth analysis could inform design of an improved governance model for strengthening the capacity of health facilities offering free maternal and child healthcare.

The study findings reveal that while decentralisation policy promoted integrated supportive supervision and monitoring of FMCHP in principle, ineffectual district FMCHP leadership resulted in poor provider performance monitoring, lack of support to frontline health workers and weak disciplinary mechanisms. These findings are consistent with results from previous studies [10,16–20]. Decentralisation should encourage more use of local decision-making and building uniform institutional capacities across health districts through effective devolution of functions, power and resources to district-level FMCHP committees. In practice, organisational culture of public service bureaucracy constrained the constitution of district-level FMCHP committees, posting and transfer, staff disciplinary mechanisms and payment of salaries through the DHBs. There is a need to reinforce district FMCHP leadership and improve accountability between district-level officials and service providers through clarity of roles and responsibilities, clear rules and procedures, provider performance monitoring and need-based resource allocation [16].

The study’s findings show that though citizen participation in management of health facilities implementing FMCHP enabled oversight of the drug revolving fund and staff attendance at work, HFCs did not play strong roles in FMCHP. Our findings are consistent with low citizen participation found in other studies [9,22–24]. In contrast, in Nepal, Burkina Faso and Kenya, HFCs were actively involved in free care schemes including financial management [10–12]. The HFCs in this study could have played stronger roles to facilitate registration of beneficiaries with primary providers in their locality, endorse provider claims and ensure that rules for spending funds reimbursed to health facilities for free care were strictly adhered to. HFCs could have also established complaint boxes, displayed service charters and created awareness about usefulness of service charters in holding providers accountable. These gaps in the roles of HFCs provide insights into the potential of citizen participation for the improvement of free health services as has been argued in a previous study [49]. It would be imperative to train and mentor HFCs and health care providers on the mandate of HFCs within the FMCHP policy to ensure that health facilities implement changes to the free care programme based on concerns raised by citizens [53].

The study findings highlight the importance of providers’ use of discretion in modifying free healthcare policy and make services unresponsive [54]. First, providers need to be paid at defined times. The findings indicate, as in several studies, that delayed reimbursement is a key implementation constraint [12,21,25]. Regular and timely reimbursement of providers could reduce chances that providers may ration free services, resume charging of user fees or impose informal payments on users. Secondly, decision makers should avoid conflictive policies that produce a contradictory incentive environment for FMCHP implementation. The study findings indicate that providers used their discretion to interpret evidence of tax payment policy, which is inconsistent with intentions of the policy but influenced how they communicated the policy to consumers. Elsewhere, health workers have employed similar discretionary power to make changes to free healthcare policies during implementation [21,25]. Conversely, consumers in this study exploited information asymmetry, evaded evidence of tax payment policy, and used SURE-P providers, which offered alternative fee-exempt maternal and child health services in some districts.

Poor remuneration in the state public health sector created a weak incentive environment for providers of free care. This finding is consistent with findings in Nigeria [24,35], but is in contrast with evidence from Sierra Leone [15]. Despite increased workload due to the free care policy, remuneration of health workers in Enugu State is at variance with nationally approved health salary scales for different cadres of health workers. This disparity in compensation, coupled with delayed payment of salaries of primary healthcare workers, is the most potent driver of poor motivation and exit of qualified health workers from Enugu’s health sector. Strategies to motivate and retain providers must take cognisance of public sector remuneration system as exemplified in Sierra Leone’s free health care initiative where salary uplift ensured that providers were adequately paid and motivated to manage increased workload without imposing informal charges on users [15].

The findings further suggest that any provider performance monitoring system that excludes district and local health authority officials is structured to fail. Even though tools for FMCHP claims were available, the preparation of claim documents was problematic at the health facility level as is the case in Burkina Faso and Burundi [10,25]. In this study, the withdrawal of district-level officials from monitoring provider performance including reporting of claims is the singular most critical gap in the task network of providers; and is consistent with evidence from Burkina Faso, where lack support from district officials limited health workers’ recording and reporting [10]. District-level officials withdrew from provider monitoring and supervision due to allegations of misappropriation of funds against them, unfavourable changes in reimbursement process and unpaid share of service charge. Consequently, providers lost the opportunity for on-the-job capacity building on and support for preparation and submission of claims.

The study findings highlight a requirement for resource allocation to providers to be need-based. The policy of remitting 70% of FMCHP service refunds directly to health facilities while potentially effective in principle, was unpredictable in practice. Also, equipment donated by development partners enabled health facilities to offer free services. However, buildings are dilapidated, health facilities lack public utilities and functional ambulances to support referrals, equipment is stacked and not tailored to the scope of services provided by facilities, which are consistent with findings of several studies [18,24,36]. Equally, the study found that donation of seed stock for the drug revolving fund and subsequent procurement through the central medical stores improved availability of quality-assured drugs in health facilities. However, some drugs donated by development partners failed to match the needs of health facilities. Some needed drugs were either expiring or not available due to delayed reimbursement, untimely replenishment of stocks and providers’ lack of funding for transport to collect drugs from the central medical store. These findings, which are consistent with evidence from several studies, partly explains health workers’ poor adherence to the free care policy and increased involvement in parallel drug supply systems [12,17,26–31].

Furthermore, the study findings indicate the need to fill gaps in staffing to meet service entitlements through recruitment and retention of appropriate cadres and mission-consistent postings and transfers especially in rural health facilities. Similarly, shortage of appropriate qualified health workers, worse in remote areas, constrained provision of maternal, neonatal and child health services in the Lao People’s Democratic Republic and three African countries, where health workers had preference for urban posts [19,36]. Though presence of SURE-P staff improved the availability of health workers, postings and transfers of health workers to less busy or urban health facilities limit responsiveness of health facilities and contribute to declining quality of care. It seems that health workers in the study setting choose less busy health facilities to enable them to cope with parallel engagements in the private sector and unauthorized continuing professional education.

Despite the existence of sanction procedures and HFCs’ monitoring of staff attendance, which could reduce rate of absenteeism among health workers, heads of health facilities are reluctant to invoke staff disciplinary measures because state-level officials do not act on recommendations of district officials, for fear of political interference in staff disciplinary processes and for sense of comradeship, similar to weak mechanisms of answerability and enforceability found in Zambia [20]. Contrastingly, Sierra Leone has shown that absenteeism could reduce drastically when a staff sanction framework is implemented within the context of a free health care initiative [15]. Improving human resource capacity will require a combination of performance-based funding to ensure that health workers are adequately motivated and enforcing sanction framework for holding providers accountable for delivery of quality services and adherence to guidelines and reporting timelines, and sanctions imposed, if specified outputs and outcomes are not delivered [15,55].

The use of case study design in this study enhanced identification of how provider factors can mediate implementation of the FMCHP. However, while sampling participants was informed by anticipated richness and relevance of information in relation to the study questions [48], the views expressed by these participants may not be generalizable to other actors. Although the study was limited to two districts, use of qualitative pilots, member check, coding framework, validation meeting, triangulation of findings from multiple sources and the full descriptions of participants’ narratives have been helpful in providing evidence that is trustworthy, relevant and has utility for decision-making.

## Conclusions

This study contributes to the scholarship and policy debate on implementation of user fee policies through its detailed analysis of Nigeria’s implementation experiences. Key lessons that should strengthen the capacity of health facilities to implement free healthcare policies include: ensure effective devolution of functions, powers and resources to district-level organisations; strengthen facility-based social accountability initiatives; harmonize concurrent and conflictive policies; ensure adequate and fair compensation of health workers, and timely reimbursement of providers; fill service delivery gaps in health facilities; and attract and retain appropriate mix of human resources. Overall, decision makers must pay attention to institutional designs and organisational practices shaping implementation of user fee removal policy at the facility level.

## Paper context

Studies examining how capacity of health facilities affect implementation of free healthcare policies in low-resource settings are limited. This study describes how context and institutional factors affect the capacity of health facilities to implement a free maternal and child healthcare programme in Nigeria. Attention to decentralisation, citizen participation, concurrent policies, task network, organisation of service delivery and human resources will enhance the institutional capacity of health facilities to offer free health care.

## Appendix 1. In-Depth Interview Guide

**Table:**

Questions	Probes
1. Could you please introduce yourself and briefly describe your work	
2. What are the main objectives and characteristics of FMCHP? 3. How have the objectives and characteristics changed overtime?	Probe for changes in service entitlement, obligations of consumers and provider payment systems. Probe for concurrent/conflictive policies
4. How does each FMCHP committee execute its roles?	Probe for discretion, authority, tools, decision space and resources to execute roles by district health boards (DHBs) and local health authorities (LHAs).
5. Are monitoring and supervision of FMCHP conducted as per guidelines?	Probe frequency, content and resources for supervision; Probe for support from DHBs and LHAs
6. What mechanisms exist to hold the MOH directly accountable to citizens for optimal implementation of FMCHP? 7. What enables or constrains citizens’ participation in FMCHP implementation at the facility level? 8. What type of consumer complaint mechanisms exist and to what extent are they being used in FMCHP/provider facilities?	Probe for HFC Alliance and citizen participation in policy and planning at facility, LHA and facility levels.
9. Who are the actors that play key roles in making decisions about FMCHP at facility level? 10. What role does each play or not play in FMCHP and why?	Ask for roles of health workers, HFCs, LHAs Secretaries and district officials in FMCHP (providing resources, provider payment and creating awareness, etc).
11. How does information flow from MOH to providers and vice versa and what enables or constrains information flow and use?	Probe for FMCHP reporting tools, data use.
12. What enables or constrains resource availability for FMCHP at health facilities?	Probe for facility funding, drugs revolving fund, infrastructure, equipment, staff attitude, availability of staff, postings and transfer, staff disciplinary procedure
13. Is needs assessment part of the resource allocation process and how are findings used?	
14. What is your general impression about FMCHP? 15. In your view, what will be the most meaningful changes to improve implementation of FMCHP?	
Thank you for your participation	

## Appendix 2. List of documents Reviewed

1. Enugu state 2013 guidelines for implementation of free maternal and u-5 child health programme. (DR1).
2. PATHS2 consultancy report on revision of FMCH policy and implementation guidelines in Enugu State. (DR2).
3. Enugu state 2013 policy on free maternal and child health services in Enugu State. (DR3).
4. Development of facility health committees to strengthen voice and accountability in Enugu State. PATHS2 Strategy Report. (DR4)
5. PATHS2 report of rapid assessment of 76 health facilities in six local government areas for implementation of essential systems and service packages. (DR5).
6. Annual performance review of Enugu state health sector. (DR6).
7. Implementation of the Enugu State free maternal and child healthcare programme: the concept, operations, challenges and prospects. Memo by former permanent secretary SMOH to Enugu State House Committee on Health (DR7).
8. Memo on FMCHP by Executive Secretary, Enugu State Planning Commission to Enugu State House Committee on Health (DR8).
9. Memo on FMCHP implementation by Facility Health Committees’ Alliance to Enugu State House Committee on Health (DR9).
10. FMCHP: Prospects, challenges and recommendations. Memo by the Chief Executive Officers of District Health Boards to Enugu State House Committee on Health (DR10).
11. Enugu State free maternal and child healthcare programme: overview, considerations and recommendations for improvement. Memo by State Health Board to Enugu State House Committee on Health (DR11).
12. An in-depth enquiry into state and local government contribution to FMCHP fund management and re-imbursement of health facilities. Memo by Local Government Service Commission to Enugu State House Committee on Health (DR12).
13. Enugu State free maternal and child healthcare programme implementation committee report, 2008. (DR13).
